# Antibodies to *Porphyromonas gingivalis* Are Increased in Patients with Severe Periodontitis, and Associate with Presence of Specific Autoantibodies and Myocardial Infarction

**DOI:** 10.3390/jcm11041008

**Published:** 2022-02-15

**Authors:** Charlotte de Vries, Guillermo Ruacho, Elin Kindstedt, Barbara Aleksandra Potempa, Jan Potempa, Björn Klinge, Pernilla Lundberg, Elisabet Svenungsson, Karin Lundberg

**Affiliations:** 1 Division of Rheumatology, Department of Medicine Solna, Karolinska Institutet and Center for Molecular Medicine, Karolinska University Hospital, 171 76 Stockholm, Sweden; guillermo.ruacho@ki.se (G.R.); elin.kindstedt@umu.se (E.K.); elisabet.svenungsson@ki.se (E.S.); 2 Public Dental Services, Folktandvården Stockholms Län AB, 113 82 Stockholm, Sweden; 3 Section for Molecular Periodontology, Department of Odontology, Umeå University, 901 85 Umeå, Sweden; pernilla.lundberg@umu.se; 4 Wallenberg Centre for Molecular Medicine, Umeå University, 901 87 Umeå, Sweden; 5 Department of Oral Immunology & Infectious Diseases, University of Louisville, Louisville, KY 40202, USA; barbara.potempa@louisville.edu (B.A.P.); jan.potempa@louisville.edu (J.P.); 6 Faculty of Biochemistry, Biophysics and Biotechnology, Jagiellonian University, 30-387 Krakow, Poland; 7 Division of Oral Diseases, Department of Dental Medicine, Karolinska Institutet, 171 77 Stockholm, Sweden; bjorn.klinge@ki.se; 8 Department of Periodontology, Faculty of Odontology, Malmö University, 205 06 Malmö, Sweden

**Keywords:** *Porphyromonas gingivalis*, autoimmunity, rheumatoid arthritis, systemic lupus erythematosus, myocardial infarction, arginine gingipains, anti-double stranded DNA antibodies, anti-citrullinated protein antibodies

## Abstract

There is accumulating data suggesting that periodontitis is associated with increased risk of systemic and autoimmune diseases, including cardiovascular disease, rheumatoid arthritis (RA) and systemic lupus erythematosus (SLE), and there is an unmet need to identify these individuals early. With the periodontal bacteria *Porphyromonas gingivalis* (*Pg*) as one of the key drivers of periodontitis, we set out to investigate whether antibodies to *Pg* virulence factor arginine gingipain (Rgp) could serve as a biomarker for periodontitis patients at increased risk of autoimmunity and systemic disease. We measured serum anti-Rgp IgG in three study populations: PAROKRANK (779 individuals with myocardial infarction (MI); 719 controls), where 557 had periodontitis, and 312 were positive for autoantibodies associated with RA/SLE; the PerioGene North pilot (41 periodontitis; 39 controls); and an SLE case/control study (101 SLE; 100 controls). Anti-Rgp IgG levels were increased in severe periodontitis compared to controls (*p* < 0.0001), in individuals positive for anti-citrullinated protein antibodies (*p* = 0.04) and anti-dsDNA antibodies (*p* = 0.035), compared to autoantibody-negative individuals; and in MI patients versus matched controls (*p* = 0.035). Our data support longitudinal studies addressing the role of anti-Rgp antibodies as biomarkers for periodontitis patients at increased risk of developing autoimmunity linked to RA and SLE, and mechanisms underpinning these associations.

## 1. Introduction

Periodontitis is an inflammatory condition in the gum mucosa, induced by microbial dysbiosis and driven by the host immune system in susceptible individuals [1]. The inflammation causes damage of supporting structures around the teeth (i.e., gingiva, periodontal ligament and bone tissue), which may result in tooth loss. Periodontitis is influenced by both genetic and environmental factors, and associates with a number of systemic inflammatory- and autoimmune conditions, including cardiovascular disease, type 2 diabetes mellitus, Alzheimer’s disease, and autoimmune diseases such as rheumatoid arthritis (RA), and systemic lupus erythematosus (SLE) [2,3,4,5,6,7]. A causative link has been proposed, but the underlying mechanisms remain to be elucidated.

One hypothesis, is that periodontitis can influence the development and maintenance of comorbidities by causing a low-grade systemic inflammation, characterized by increased levels of pro-inflammatory factors such as interleukin (IL) 1, IL-6, and C-reactive protein (CRP), that lead to metabolic and inflammatory alterations [7,8]. Furthermore, successful periodontal treatment attenuates systemic inflammatory markers and may reduce markers of comorbid conditions. Accordingly, recent work from d’Aiuto and colleagues shows that periodontal treatment effectively cause a reduction of glycosylated haemoglobin (HBA1c) in diabetes patients, supporting a causal relationship [9].

Several of these inflammatory markers are not specific for periodontitis, and there is currently no established specific serological biomarker available for diagnosing periodontitis, or to define subsets of periodontitis patients, for example those that may be at increased risk of developing systemic disease. A number of studies have analysed antibody responses to oral pathogens in this context, including *Porphyromonas gingivalis* (*Pg*), a Gram-negative anaerobic periodontal bacterium known as one of the key pathogens driving periodontitis [10]. *Pg* expresses virulence factors such as lipopolysaccharide (LPS), capsules, fimbriae and gingipains, involved in transforming the symbiotic microbiota into a dysbiotic proinflammatory microbial community causing disease [11,12]. Gingipains, which are extracellular cysteine proteases, are the most potent of the virulence factors, capable of degrading host proteins causing tissue destruction and evasion or subversion of host immune responses [13,14]. Recently, a thorough investigation of antibody responses to different *Pg*-derived antigens showed that serum IgG levels in periodontitis patients were highest against these gingipains, specifically the arginine gingipains (Rgp) [15].

In a small pilot study, we have previously shown an increased serum antibody response to Rgp in periodontitis patients compared to periodontally healthy individuals. We have also reported significantly elevated anti-Rgp IgG levels in patients with RA, up to 10 years before onset of RA symptoms, compared to population controls, with highest levels detected in RA patients positive for anti-citrullinated protein antibodies (ACPA) [16,17]. This is of particular interest since *Pg* is the only pathogen known to be able to citrullinate proteins [18]. Hence it has been suggested that *Pg* may have a central role in linking periodontitis to RA, by generating citrullinated antigens in the inflamed gum mucosa. Such antigens could trigger loss of tolerance and systemic ACPA production, subsequently causing RA via the formation of ACPA-immune complexes in synovial joints [19,20].

Elevated anti-*Pg* IgG levels in RA versus controls have been confirmed in a meta-analysis [21], and a number of studies support an association between periodontitis/*Pg* and the autoimmune ACPA response [22,23,24,25,26,27], including a report demonstrating presence of ACPA in crevicular fluid of periodontitis patients [28]. A possible link between periodontitis and SLE has also been investigated, and a significant association was identified in a meta-analysis [5]. In addition, oral dysbiosis has been reported in SLE [29], and antibodies to oral bacteria, including *Pg*, were reported to be increased in anti-dsDNA antibody positive SLE patients [30].

With the present study, we set out to confirm the link between elevated anti-Rgp IgG levels and periodontitis in a larger cohort, and to determine whether anti-Rgp IgG identifies a specific subset of periodontitis patients. Additionally, to increase our understanding of the relationship between *P. gingivalis* and autoimmunity, we examined Rgp IgG in relation to presence of 15 RA- and SLE associated autoantibodies, including ACPA and anti-dsDNA antibodies. Moreover, by taking advantage of the well-characterised PAROKRANK study comprising 805 individuals with a first myocardial infarction (MI) and 805 matched controls, where detailed periodontal diagnostics is available, we also investigated the association between anti-Rgp IgG, autoantibodies and MI.

## 2. Materials and Methods

### 2.1. Study Design

**Figure 1:**
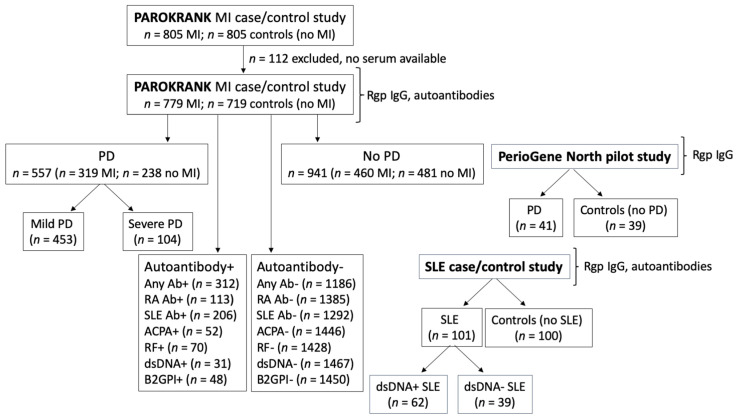
Flowchart describing the study design. Serum samples from three separate studies (PAROKRANK, the PerioGene North pilot study and an SLE case/control study) were analysed for anti-Rgp IgG levels, and presence of different autoantibodies (PAROKRANK and the SLE case/control study only). Anti-Rgp IgG levels were compared between different subgroups: MI versus non-MI controls; PD versus non-PD controls (and between no, mild and severe PD); autoantibody positive versus autoantibody negative; and SLE versus non-SLE controls. Additional autoantibodies, not included in the flowchart, were also analysed. *n* = number; MI = myocardial infarction; PD = periodontitis; SLE = systemic lupus erythematosus; Ab = autoantibody; RA = rheumatoid arthritis; ACPA = anti-citrullinated protein antibody; dsDNA = double stranded DNA; RF = rheumatoid factor; B2GPI = beta-2-glycoprotein.

In order to evaluate antibodies to arginine gingipains as potential biomarkers for periodontitis subsets, we have measured anti-Rgp IgG in serum samples from three separate study populations, described in detail below, and compared antibody levels in: (i) individuals with periodontitis versus no periodontitis (and in relation to periodontitis severity); (ii) patients with MI versus matched controls; (iii) patients with SLE versus non-SLE controls; and in (iv) individuals with RA and/or SLE -associated autoantibodies versus autoantibody negative individuals. See Figure 1 for a flowchart describing the study design, including number of individuals per subgroup analysed.

### 2.2. Study Populations

We included 1498 individuals from the PAROKRANK study [31], a Swedish multicentre case-control study, comprising patients < 75 years of age that were hospitalized for a first myocardial infarction. Controls were individually matched to cases based on age, sex and postal code area. Each study participant underwent a physical examination at the cardiology department and an extensive dental examination, including radiographic examination, within 6 to 10 weeks after inclusion. Bleeding on probing (BOP) was measured at four sites per tooth, and BOP index was calculated based on the total number of measured sites. BOP was categorized in grade 0, 1 or 2, with a BOP index of 0–9.9%, 10.0–29.9% and ≥30%, respectively. Based on the radiographical examination (mean bone loss of all teeth), participants were classified into three groups: no periodontitis (≥80% remaining bone), mild to moderate periodontitis (66–79% remaining bone), and severe periodontitis (<66% remaining bone) [31] Individuals were excluded when having had a prior myocardial infarction or prior heart valve replacement.

From the PerioGene North case-control study, we have selected 41 patients with severe periodontitis (here defined as having periodontal damage with at least 50% of teeth showing bone loss exceeding >1/3 of the root length, BOP at >20% of the periodontal pockets and having >15 remaining teeth), and 39 periodontally healthy individuals (defined as having no signs of periodontal attachment loss, probing pocket depth < 4 mm at all sites, and being ≥35 years with ≥24 remaining teeth) [32]. BOP was measured at six different sites per tooth. Exclusion criteria were: (i) antibiotic usage or periodontal treatment in the previous three months, (ii) pregnancy or lactancy, or (iii) having any other known disease or ongoing therapy with anti-inflammatory drugs.

From a cohort of 546 consecutive SLE patients treated at the rheumatology clinic, Karolinska University Hospital, Stockholm, Sweden, and 322 age, sex and residential area matched controls [33], we randomly selected 101 patients and 100 controls (Supplementary Table S1). SLE patients were ≥18 years old and fulfilled ≥4 of the American College of Rheumatology (ACR) 1982 revised classification criteria for SLE [34]. Periodontal status was not available for the SLE cohort.

### 2.3. Anti-Rgp IgG ELISA

Serum samples were analysed by an in-house enzyme-linked immunosorbent assay (ELISA) for the presence of anti-Rgp IgG, as previously described [16,35]. In short, 96-well Nunc MaxiSorp ELISA plates were coated with 50 µL coating solution, containing 5 µg/mL RgpB protein in coating buffer (50 mM carbonate buffer, pH 9.5), overnight at 4 °C. C-terminal hexahistidine-tagged RgpB protein was purified from growth medium of genetically modified *P. gingivalis* strain W83, by affinity chromatography on Ni-Sepharose as previously described [36]. Plates were washed three times with phosphate buffered saline (PBS)-Tween (0.05%) and blocked with 2% bovine serum albumin (BSA) in PBS at room temperature (RT) for 2 h. Serum samples were diluted 1:400 in RIA buffer (1% (*w*/*v*) BSA, 350 mM NaCl, 10 mM Tris-HCl (pH 7.6), 1% (*v*/*v*) Triton X-100, 0.5% (*w*/*v*) Na-deoxycholate, 0.1% (*w*/*v*) SDS) and incubated for 1.5 h at RT. After washing, peroxidase-conjugated mouse anti-human IgG (diluted 1:10 000 in RIA buffer) was added for 1.5 h at RT. Plates were washed again, and antibody binding detected by addition of tetramethylbenzidine substrate for 15–18 min. The reaction was stopped by addition of 1 M H_2_SO_4_, and absorbance measured at 450 nm. A standard curve, based on a pool of Rgp IgG-positive sera in serial dilution, was used to calculate antibody titres in arbitrary units (AU). All serum samples were analysed in duplicates, and a blank was included on all plates.

### 2.4. Detection of Autoantibodies

Autoantibody measurements were performed by routine analysis at the SWEDAC (www.swedac.se, accessed on 9 January 2022) accredited Clinical Chemistry and Immunology Laboratories at the Karolinska University Hospital, Stockholm, Sweden. Antinuclear antibodies (ANA) were analysed by indirect immunofluorescence on HEp-2 cells (Immunoconcepts, Sacramento, CA, USA). Antibodies to specific nuclear antigens (dsDNA, SSA-Ro52, SSA-Ro60, SSB, Sm, RNP) and phospholipid related antigens (cardiolipin IgG, IgM, IgA and β2-glycoprotein1 IgG, IgM, IgA) as well as ACPA (detected as anti-cyclic citrullinated peptide 2 (CCP2) IgG) were analysed by multiplexed bead technology (Luminex) using the BioPlex 2200 system (Bio-Rad, Hercules, CA, USA). Rheumatoid factor (RF) (non-class specific) was measured by nephelometry. In the SLE cohort, IgM RF was analysed by fluorescence enzyme immunoassay using the EliA system on a Phadia250 equipment (Thermo Fisher Scientific, Uppsala, Sweden).

### 2.5. Statistical Methods

Data analyses were performed using R, version 4.1.1 (R Foundation for statistical computing, Vienna, Austria) and Statistical Package for Social Sciences (SPSS), version 24 or 26 (IBM Corporation, Armonk, NY, USA), JMP, version 13.0 (SAS Institute Inc., Cary, NC, USA), and Prism, version 8. Continuous variables are presented as median, with the 10th–90th percentile range (box plot graphs) or with min and max values (tables), and categorical variables as frequencies. Group comparisons were performed using Students *t*-test, Kruskal-Wallis, Mann-Whitney, Pearson’s chi-square or Fisher exact tests when appropriate. Statistical significance was set at *p* ≤ 0.05.

To determine sensitivity and specificity for anti-Rgp IgG, we calculated the accuracy to discriminate periodontitis patients from controls, using area under the receiver-operating characteristic curves (AUROC) with 95% confidence intervals (CI). Youden’s J statistic was used to determine the highest sensitivity and specificity.

Matched analyses (where we matched individuals based on age, sex and smoking) were performed using coarsened exact matching with the MatchIt package in R [37].

## 3. Results

### 3.1. Increased Anti-Rgp IgG Levels Associate with Periodontitis Severity

In PAROKRANK, 557 out of 1498 individuals were defined as having periodontitis according to the degree of bone loss on panoramic X-ray [38]. As expected, BOP and presence of deep pockets were significantly higher in the periodontitis group (Table 1). Well-known risk factors for periodontitis were also significantly increased in the periodontitis subset. Thus, periodontitis patients were older, more frequently smokers, and elevated HbA1C levels suggest diabetes was more common. Having had a first myocardial infarction was also more common among periodontitis patients, as we have recently reported [31]. The proportion of men was significantly lower in the periodontitis group.

**Figure 2:**
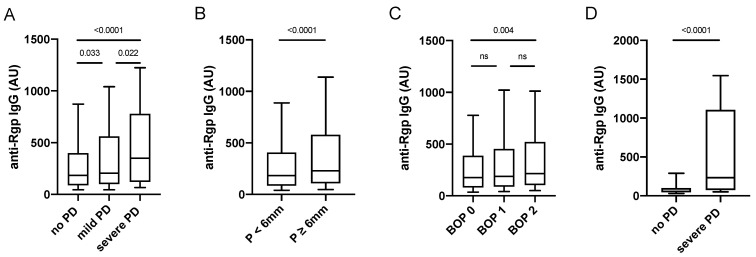
Anti-Rgp antibody levels are increased in severe periodontitis. Rgp IgG levels in PAROKRANK, based on periodontitis status (**A**), pocket depth (**B**), and BOP (**C**). Rgp IgG levels in PerioGene North, based on periodontitis status (**D**). Median AU values are shown as horizontal lines, whiskers indicate 10th–90th percentile; *p*-values show differences between groups. PD = periodontitis; P = pocket depth; BOP = bleeding on probing; ns = not significant.

When analysing anti-Rgp antibodies in PAROKRANK, we detected significantly increased levels in individuals with periodontitis versus non-periodontitis controls, and when dividing patients based on periodontitis severity, we found highest Rgp IgG levels in the group with severe periodontitis, defined as <66% of remaining bone on panoramic X-ray (Figure 2A). In line with this observation, elevated Rgp IgG levels were also associated with the individual dental measurements BOP and presence of periodontal pockets ≥ 6 mm (Figure 2B,C). Data remained significant, when matching periodontitis cases to non-periodontitis controls, based on age, sex and smoking (data not shown).

We also analysed a smaller number of serum samples from a separate cohort, comprising 41 patients with severe periodontitis, selected from the PerioGene North cohort [32] based on having active disease, and 39 periodontally healthy individuals (Table 2). Here, markedly increased Rgp IgG levels could be confirmed in patients with severe periodontitis versus periodontally healthy (Figure 2D).

**Figure 3:**
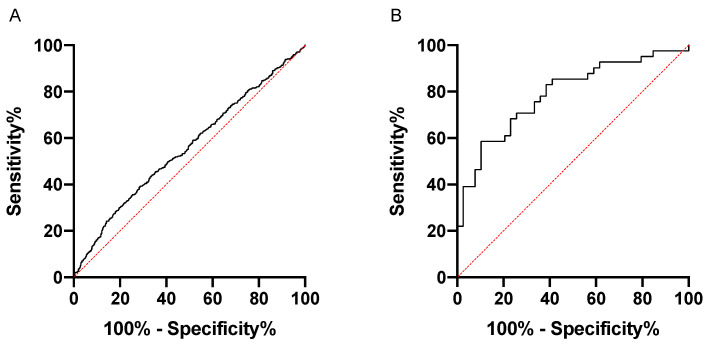
Rgp IgG discriminates periodontitis patients from periodontally healthy in PerioGene North but not in PAROKRANK. ROC curve analyses based on Rgp IgG levels in PAROKRANK (**A**) and PerioGene North (**B**).

To further evaluate Rgp IgG as a biomarker for periodontitis, we performed ROC curve analyses to determine the sensitivity and specificity. When using the PerioGene North Rgp IgG data, we could demonstrate a sensitivity of 58.4%, with a specificity of 89.7% (AUROC: 0.7880; 95% CI: 0.6883–0.8876), while the PAROKRANK Rgp IgG data could not be used to efficiently separate periodontitis from non-periodontitis study participants (AUROC: 0.5578; 95% CI: 0.5274 to 0.5883) (Figure 3).

### 3.2. Increased Anti-Rgp IgG Levels Associate with Presence of ACPA and Anti-dsDNA Antibodies

**Figure 4:**
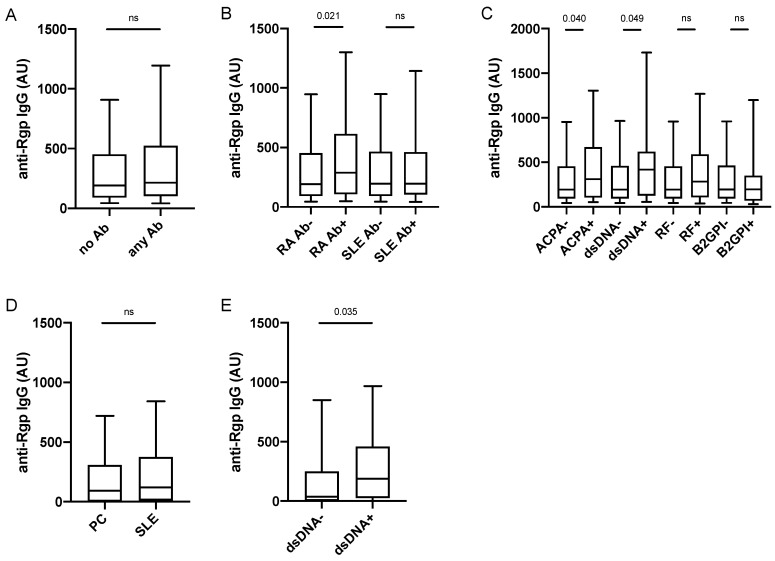
Anti-Rgp IgG levels are increased in ACPA+ and dsDNA+ individuals. Rgp IgG levels in PAROKRANK, based on presence/absence of any autoantibody (**A**), RA- or SLE-associated autoantibodies (**B**), and ACPA, anti-dsDNA antibodies, RF and anti-B2GPI antibodies (**C**). Rgp IgG levels in 101 SLE patients and 100 controls (**D**) and in SLE patients with (*n* = 62) or without (*n* = 39) anti-dsDNA antibodies (**E**). Median AU values are shown as horizontal lines, whiskers indicate 10th–90th percentile. *p*-values show differences between groups. AU = arbitrary units; Ab = autoantibody; ACPA = anti-citrullinated protein antibody; dsDNA = double stranded DNA; RF = rheumatoid factor; B2GPI = beta-2-glycoprotein; ns = not significant, RA = rheumatoid arthritis; SLE = systemic lupus erythematosus; PC = population controls.

We next screened PAROKRANK for presence of autoantibodies, including ACPA, RF, ANA sub-specificities and anti-PL autoantibodies (Supplementary Table S2) in order to examine their relationship with Rgp IgG. Twenty percent (*n* = 312) of PAROKRANK study participants were positive for at least one of the investigated autoantibodies. When analysing Rgp IgG levels in this autoantibody-positive subset, compared to the autoantibody-negative subset, we did not detect any significant difference (Figure 4A). However, when PAROKRANK participants were divided based on presence/absence of RA-associated autoantibodies only (i.e., ACPA and RF), present in 7.5% (*n* = 113), we observed significantly elevated Rgp IgG levels in the antibody-positive subset (Figure 4B). This was not seen when PAROKRANK participants were divided based on presence/absence of SLE-associated autoantibodies only (listed in Supplementary Table S2), present in 14% (*n* = 206).

When focusing the analysis on individual autoantibodies, we could confirm our previous data [16], demonstrating increased Rgp IgG levels in the ACPA-positive versus the ACPA-negative subset (Figure 4C). Interestingly, we also detected significantly increased Rgp IgG levels in individuals positive for anti-dsDNA antibodies, while no difference was found for any of the other 14 autoantibodies investigated, including RF and anti-B2GPI IgG, corresponding to the two most frequently detected autoantibodies in PAROKRANK. There was only one individual double-positive for ACPA and anti-dsDNA antibodies, while 10 were double-positive for ACPA and RF, and two were double-positive for RF and anti-dsDNA antibodies. Data were confirmed in matched analyses, where each autoantibody-positive individual was matched on age, sex and smoking to autoantibody-negative individuals (data not shown).

Our observations in PAROKRANK were confirmed in a separate SLE case-control study (described in Supplementary Table S1). Anti-Rgp IgG levels did not differ between SLE patients and controls, while they were increased in anti-dsDNA antibody-positive compared to anti-dsDNA antibody-negative SLE patients (Figure 4D,E). None of the other investigated SLE-associated autoantibodies (listed in Supplementary Table S2) or RF, had an impact on Rgp IgG levels, nor did total IgG or total IgM levels (data not shown). Moreover, we could not detect any associations between Rgp IgG levels and SLE-associated clinical activity scores or any of the other tested clinical variables (listed in Supplementary Table S3).

### 3.3. Increased Anti-Rgp IgG Levels Associate with Myocardial Infarction

**Figure 5:**
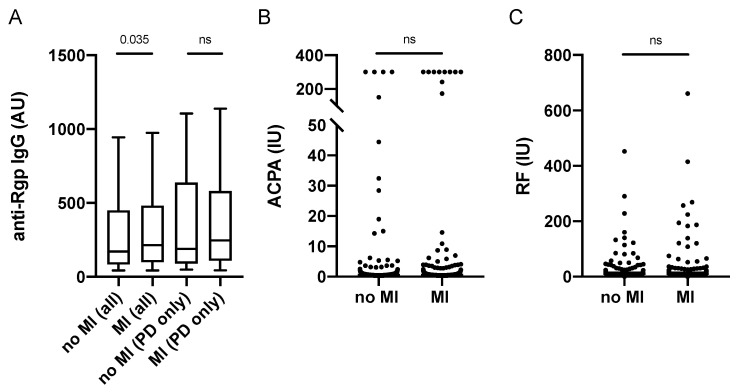
Anti-Rgp antibodies, ACPA and RF levels in relation to myocardial infarction in PAROKRANK. Rgp IgG levels based on MI and periodontitis status (**A**). ACPA (**B**) and RF (**C**) levels based on MI status. Median AU values are shown as horizontal lines, whiskers indicate 10th–90th percentile; *p*-values show differences between groups. AU = arbitrary units, IU = international units, MI = myocardial infarction; PD = periodontitis, ACPA = anti-citrullinated protein antibodies; RF = rheumatoid factor; ns = not significant.

Since the PAROKRANK study was first established to investigate the link between periodontitis and myocardial infarction (MI), and our previous PAROKRANK report has demonstrated increased prevalence of periodontitis in MI patients versus controls [31], we next analysed Rgp IgG levels in relation to MI status. We found significantly increased levels in patients who were affected with a first MI, compared to matched controls (Figure 5A). However, Rgp IgG levels did not differ when only periodontitis patients (with and without MI) were analysed, though we did detect an enrichment of individuals with severe periodontitis in the MI group (22.9%) versus the control group (13.4%), *p* = 0.0068.

We have previously reported significantly higher levels of anti-PL antibodies in MI versus controls, which was not seen for any other SLE-associated autoantibody, including anti-dsDNA IgG [39]. Here we can now show that there is also no difference in RA-associated autoantibody levels (i.e., ACPA and RF) between MI patients and controls in PAROKRANK (Figure 5B,C).

## 4. Discussion

There is accumulating data suggesting that patients with periodontitis are at an increased risk of developing systemic and autoimmune diseases [3,4,5,6]. The underlying mechanisms are to a large extent unknown, and there is an unmet need to identify these individuals at an early stage. The use of serological biomarkers in this context would be desirable. Since previous studies from our group and others suggest a role for periodontal microbes, we focused our investigation on the antibody response towards a key pathogen in periodontitis, *Porphyromonas gingivalis*.

In the large PAROKRANK study, we here demonstrate an increased antibody response to the *Pg* virulence factor arginine gingipains in patients with periodontitis versus controls. These results were confirmed in a selected group of periodontitis patients and controls from the PerioGene North cohort. Notably, the highest levels were detected in (i) severe periodontitis, (ii) in the presence of ACPA and anti-dsDNA antibodies, and (iii) in patients who had suffered from a first myocardial infarction.

We can thus confirm our previous results [16], demonstrating a link between elevated anti-Rgp IgG levels and periodontitis, and further demonstrate a positive association with periodontitis severity, measured as bone loss, bleeding on probing and pocket depth. Moreover, in the smaller PerioGene North pilot cohort, Rgp IgG showed a moderate to high sensitivity and specificity for (severe) periodontitis, clearly discriminating patients from controls. This finding is in line with recent data from Hirai and colleagues, on antibodies reactive with the N-terminal part of RgpA, encompassing the catalytic domain which shares >98% sequence identity with RgpB used in our study [15].

On the other hand, in PAROKRANK, Rgp IgG did not clearly separate the periodontitis affected from the non-affected individuals, indicating that it might not be a suitable serological biomarker for periodontitis per se. The discrepant results between PAROKRANK and PerioGene North, could be related to the stricter definition of periodontally healthy in PerioGene North, compared to PAROKRANK. In both the PerioGene North cohort, and in the study from Hirai et al., the control groups were young (median age 44 and 34 years, respectively) and truly periodontally healthy, while in PAROKRANK, study participants were older (median age 64 years) and signs of periodontal inflammation (e.g., BOP and deep pockets) were present also among individuals classified as not having periodontitis.

Age is one of the best-known risk factors for periodontitis, and it is far less common to find individuals who have not been affected by periodontitis in an older population; nearly 50% of 50–64 year olds are expected to have some form of periodontitis [40]. According to the most recent classification criteria, periodontitis is defined as having radiographic approximal bone loss or clinical attachment loss at more than two non-adjacent teeth that cannot be ascribed to non-periodontal causes [41]. In the case of the PAROKRANK study, to our current knowledge, the number of individuals having some form of periodontitis might have been underestimated. Periodontitis was diagnosed based solely on radiographic images, which may not have been the best way to estimate bone loss [42,43].

Other reasons as to why anti-Rgp antibody levels did not accurately match with the periodontal status in PAROKRANK could be that the dysbiotic bacteria in the oral biofilm, contributing to periodontitis, did not include *Pg* [44], or Rgp IgG levels may also have reflected past exposure to *Pg*, rather than current periodontal infection [45]. Longitudinal studies, starting at a young age, are warranted to further investigate the potential of Rgp IgG as a relevant biomarker for periodontitis per se, and detailed matched periodontitis case-control studies are needed to further evaluate anti-Rgp IgG in relation to clinically different periodontitis subsets.

The main focus of the present study was the relationship between Rgp IgG and autoantibodies. We have previously reported a link between Rgp IgG and ACPA in a large RA case-control study [16] and these observations were now confirmed in PAROKRANK. Interestingly, despite a low frequency (2.1%) of anti-dsDNA antibodies we observed a positive association with elevated anti-Rgp IgG levels in PAROKRANK, and these findings were confirmed in a separate SLE cohort (61.4% dsDNA+). Our data are in line with a previous study, demonstrating increased antibody levels to *Pg* as well as to other periodontal pathogens in anti-dsDNA-positive SLE patients [30]. However, in contrast to the other study [30], we did not observe any association between anti-*Pg* antibodies and SLE per se or SLE disease activity. This discrepancy may be related to statistical power, since the other study was larger than ours (*n* = 303 versus *n* = 101), or to the type of SLE patients included, as SLE is a notoriously heterogenous disease [46]. We found no association between elevated anti-Rgp IgG levels and presence of autoantibodies in general, including RF, ANA and aPL antibodies, in any of the two cohorts investigated. Notably though, as already mentioned, the SLE case-control study was small, and in PAROKRANK, several of these autoantibodies were present at very low frequencies, which may have affected the statistical power of the analyses.

We speculate that the association between Rgp IgG and ACPA/dsDNA may be related to the nature of the autoantigens, in that both dsDNA and citrullinated proteins are exposed to the immune system during neutrophil extracellular trap (NET) formation. Arginine gingipains derived from *Pg* are efficient triggers of NETosis, through cleavage of Protease Activated Receptor-2 on the neutrophil surface [47]. Interestingly, *Pg*-induced NETs have impaired antibacterial activity, and are instead thought to support bacterial growth [48]. Hence, periodontitis is associated with extensive accumulation of NETs, which contribute to increased inflammation and tissue destruction, and the release of dsDNA and citrullinated histones. When exposed to the immune system in the bacteria-rich inflammatory periodontium, these antigens may trigger loss of tolerance and production of ACPA or anti-dsDNA autoantibodies in genetically susceptible individuals.

Concerning the ACPA response, it has also been suggested that *Pg* may be more directly involved in generating citrullinated autoantigens, by the combined actions of Rgp and the *Pg* peptidyl arginine deiminase enzyme *P.*PAD, where Rgp cleaves polypeptides at arginine residues, following conversion of arginine to citrulline by *P.*PAD [19,20]. *P*.PAD has the capacity to citrullinate RA candidate autoantigens [49,50], and presence of multiple citrullinated proteins have been confirmed in the inflamed periodontium [51]. Moreover, the first clinical ACPA test detected antibodies in RA sera that bound citrullinated filaggrin in buccal mucosa [52], supporting a link between the oral mucosa/*Pg*/periodontitis and ACPA + RA.

Our study also showed elevated Rgp IgG levels in patients who were diagnosed with a myocardial infarction, when compared to matched controls. This observation is in line with our previous data from PAROKRANK, demonstrating a higher frequency of periodontitis in MI patients versus controls [31], and other studies reporting an association between periodontitis and cardiovascular disease [53]. Interestingly, *Pg* DNA has been detected in coronary arteries and veins [54], and it has been suggested that oral pathogens may colonize sites outside of the oral cavity, and that such distant colonisation may trigger an inflammatory response. Notably though, we did not observe higher Rgp IgG levels in MI patients when stratified for periodontitis, even though patients with severe periodontitis were enriched among MI patients. Hence, in our study, Rgp IgG could not identify MI patients within the periodontitis group, and shared risk factors and/or shared inflammatory pathways may be more likely explanations to the association between periodontitis and MI. Moreover, patients with RA and SLE have an increased risk for cardiovascular disease [55,56], and a positive association between anti-PL antibodies and MI has previously been reported in PAROKRANK [39]. However, we found no support for an association between other SLE or RA-related autoantibodies and MI [57]. This finding contradicts a recent report demonstrating an association between elevated ACPA levels and increased risk for acute coronary syndrome in a large (*n* = 2814) RA cohort, and may be a statistical power issue related to the low frequency of ACPA in PAROKRANK (7.5%) compared to the RA cohort (65%).

Studying autoantibodies in a study such as PAROKRANK, which was designed for another purpose (i.e., to investigate the association between periodontitis and MI), introduced the problem with low frequencies of the studied autoantibodies, and for some autoantibodies the frequencies were even too low to perform the analyses. Still, for the association between elevated Rgp IgG levels and ACPA-positive RA, the data confirms our previous results from a large RA cohort [16], and for anti-dsDNA antibodies, the initial observation was confirmed in a separate SLE study population.

Our study has some additional limitations, including the lack of clinical data for RA and SLE diagnoses in PAROKRANK. As a result, our analyses were based on presence of autoantibodies as surrogates for autoimmune disease. When interpreting these data, one has to keep in mind that autoantibodies often precede clinical onset [58,59], and can be present in healthy individuals. Moreover, even though PAROKRANK comprises matched cases and controls, matching was performed based on cases having MI, not periodontitis Hence, some level of bias may have been introduced due to confounding factors, when comparing individuals with periodontitis with those without periodontitis. Smoking for example, is a risk factor for myocardial infarction, periodontitis and ACPA-positive RA. However, we have previously shown that smoking is associated with lower Rgp IgG levels, rather than higher [16]. In addition, importantly, we performed additional matched (age, sex and smoking) analyses, confirming our findings.

## 5. Conclusions

To summarise, our study demonstrates that increased levels of antibodies to *P. gingivalis* arginine gingipains associate with periodontitis severity, myocardial infarction, and with the occurrence of ACPA and dsDNA antibodies. While we cannot provide data supporting the use of anti-Rgp IgG as a biomarker for periodontitis in a general population, this antibody could be valuable for the identification of clinical subgroups, something that should be investigated in larger, well-matched case-control studies. With regard to elevated anti-Rgp IgG levels in MI patients versus matched controls, the large PAROKRANK cohort gives significant power, and strengthens our previous finding, in support of chronic periodontal infection as a risk factor for myocardial infarction [31]. However, anti-Rgp IgG does not seem to identify MI within the group of periodontitis patients, hence could not serve as a biomarker in this sense. Our data on anti-Rgp IgG in relation to ACPA and dsDNA antibodies on the other hand provide a strong rationale for further studies addressing the role of Rgp IgG as a biomarker for periodontitis patients at increased risk of developing specific autoimmunity linked to RA and SLE, as well as experimental studies exploring the molecular mechanisms underpinning these associations.

## Figures and Tables

**Table 1:** Baseline characteristics and clinical dental variables in PAROKRANK, based on periodontal status.

	Periodontitis (*n* = 557)	No Periodontitis (*n* = 941)	*p* Value ^1^
MI patient, *n* (%)	319 (57%)	460 (49%)	0.002
Male sex, *n* (%)	435 (78%)	783 (83%)	0.017
Smoking, ever, *n* (%) ^2^	408 (74%)	473 (51%)	<0.001
Age, median years (range)	66 (40–75)	62 (28–75)	<0.001
HbA1C, median mmol/mol (range)	39 (26–117)	38 (20–94)	<0.001
BOP index, median % (range) ^3^	25 (0.9–100)	21 (0.9–100.)	<0.002
Boneloss, median % (range)	24.5 (20.0–100)	15.4 (6.4–19.9)	<0.001
Presence of pockets ≥ 6 mm, *n* (%)	251 (55.5%)	309 (26.7%)	<0.001

^1^ *p*-values show differences between periodontitis and no periodontitis subgroups. ^2^ Smoking, ever includes current and former smokers. ^3^ 19 individuals were excluded from the analysis, due to missing data. *n* = number; MI = myocardial infarction; HbA1C = haemoglobin A1c; BOP = bleeding on probing.

**Table 2:** Baseline characteristics and clinical dental variables in PerioGene North.

PerioGene North	Periodontitis (*n* = 41)	No Periodontitis (*n* = 39)	*p* Value ^1^
Male sex, *n* (%)	21 (51.2)	15 (38.5)	ns
Smoking, ever, *n* (%)	29 (70.7)	9 (23.1)	<0.001
Age, median years (range)	55 (28–76)	44 (35–63)	<0.001
BOP index, median % (range)	35 (8–100)	6 (0–27)	<0.001
Number of teeth with bone loss, median (range)	18 (7–29)	0	<0.001
Number of teeth with pocket ≥ 4 mm, median (range)	21 (3–28)	0	<0.001

^1^ *p*-values show differences between periodontitis and no periodontitis subgroups. *n* = number; ns = not significant; BOP = bleeding on probing.

## Data Availability

All data generated during this study are included in this published article, and in its supplementary information files, or are available from the corresponding author on reasonable request. The PAROKRANK dataset is deposited at Karolinska Institutet, Stockholm, Sweden.

## Supplementary Materials

The following are available online at https://www.mdpi.com/article/10.3390/jcm11041008/s1: Table S1: Baseline characteristics in the SLE cohort, Table S2: Autoantibodies in PAROKRANK and in the SLE cohort, Table S3: Clinical variables in the SLE cohort.
