# Supplementary material for: Antibodies to Porphyromonas gingivalis Are Increased in Patients with Severe Periodontitis, and Associate with Presence of Specific Autoantibodies and Myocardial Infarction
Source: J Clin Med. 2022 Feb 15;11(4):1008. doi: 10.3390/jcm11041008 (PMC8875626; doi:10.3390/jcm11041008)
Supplement: Supplementary file 1 [file jcm-11-01008-s001.zip › Supplementary table 1_deVries.pdf]

Supplementary Table S1. Baseline characteristics in the SLE cohort

	SLE patients (n=101)	Controls (n=100)	P value [1]
Male sex, n (%)	8 (8)	6 (6)	ns
Smoking, ever, n (%)	56 (55)	52 (52)	ns
Age, years (range)	44 (18-77)	50 (20-82)	0.0058

[1] P-values show differences between SLE patients and controls. N = number; ns = not significant. Smoking, ever includes current and former smokers.
