# Supplementary material for: Antibodies to Porphyromonas gingivalis Are Increased in Patients with Severe Periodontitis, and Associate with Presence of Specific Autoantibodies and Myocardial Infarction
Source: J Clin Med. 2022 Feb 15;11(4):1008. doi: 10.3390/jcm11041008 (PMC8875626; doi:10.3390/jcm11041008)
Supplement: Supplementary file 1 [file jcm-11-01008-s001.zip › Supplementary table 2_deVries.pdf]

Supplementary Table S2. Autoantibodies in PAROKRANK and in the SLE cohort

AutoAb	PAROKRANK (n=1498) n (%) [1]	SLE patients (n=101) n (%)
**CCP2 IgG**	52 (3.5)	*NA*
**RF**	70 (4.7)	28 (27.7)
**dsDNA**	31 (2.1)	62 (61.4)
**Sm**	1 (0.0)	21 (20.8)
**SmRNP**	4 (0.3)	30 (29.7)
**SSA Ro52**	12 (0.8)	35 (34.7)
**SSA Ro60**	12 (0.8)	44 (43.6)
**SSB**	18 (1.2)	24 (23.8)
**RNP 68**	4 (0.3)	15 (14.9)
**B2GP1 IgA**	14 (0.9)	13 (12.9)
**B2GP1 IgG**	48 (3.2)	21 (20.8)
**B2GP1 IgM**	31 (2.1)	4 (4.0)
**CL IgA**	14 (0.9)	12 (11.9)
**CL IgG**	52 (3.5)	20 (19.8)
**CL IgM**	34 (2.3)	6 (5.9)
**ANA**	*NA*	98 (97.0)
**Centromere**	*NA*	3 (3.0)
**Ribosomal P protein**	*NA*	4 (4.0)
**Nucleosome**	*NA*	50 (49.5)

[1] Numbers (n) and frequencies (%) of different autoantibodies are shown. CCP2 = cyclic citrullinated peptide 2, RF = rheumatoid factor, dsDNA = double stranded DNA, Sm = Smith, SmRNP= Smith ribonucleoprotein, SSA = Sjögren's-syndrome-related antigen A, SSB = Sjögren's-syndrome-related antigen B, B2GP1 = β2-glycoprotein1, CL = cardiolipin, ANA = anti-nuclear antibodies, NA = not analysed.
