# Supplementary material for: Antibodies to Porphyromonas gingivalis Are Increased in Patients with Severe Periodontitis, and Associate with Presence of Specific Autoantibodies and Myocardial Infarction
Source: J Clin Med. 2022 Feb 15;11(4):1008. doi: 10.3390/jcm11041008 (PMC8875626; doi:10.3390/jcm11041008)
Supplement: Supplementary file 1 [file jcm-11-01008-s001.zip › Supplementary table 3_deVries.pdf]

Supplementary Table S3. Clinical variables in the SLE cohort

**CLINICAL VARIABLES[1]:**
Malar rash
Discoid lesions
Photosensitivity
Oral ulcers
Arthritis
Pleuritis
Pericarditis
Serositis
Nephritis
Psychosis
Seizures
Leukopenia
Lymphopenia
Thrombocytopenia
Haemolytic anemia

[1] *Clinical variables, defined according to the revised ACR criteria for SLE, from 1982 (*Tan *et al*, The 1982 revised criteria for the classification of systemic lupus erythematosus. Arthritis Rheum. 1982;25:1271-7).
